# Benign chondroid syringoma of the orbit: a rare cause of exophtalmos

**DOI:** 10.1186/1746-160X-8-8

**Published:** 2012-03-08

**Authors:** Hatim Belfquih, Brahim El mostarchid, Mohamed Oukabli, Ali Akhaddar, Mohammed Boucetta

**Affiliations:** 1 Departments of Neurosurgery and Anatomopathology Mohammed V Military Teaching Hospital, Mohammed V Souissi University, Rabat, Morocco; 2 Anatomopathology Mohammed V Military Teaching Hospital, Mohammed V Souissi University, Rabat, Morocco; 3 N 273, Hay Al Khiam III Temara, Morocco

**Keywords:** Chondroid syringoma, Exophtalmos, Intraorbital tumor, Lateral orbitotomy

## Abstract

Chondroid syringoma (CS) of the orbit is an extremely rare benign neoplasm. To the best of our knowledege, this is the second case reported in the english litérature.

We report a case of a 41-year-old woman with orbital CS. This tumor developed slowly over 8 years causing indolor, no axil, exophtalmos of the left eye. Computed tomography demonstrated an isodense intraorbital tumor with homogeneous enhancement without bony erosion. On Magnetic resonance imaging the tumor was isointense on T1-weighted imaging, slightly hyper intense on T2-weighted imaging, and enhanced after Gadolinium administration. The patient was operated via left lateral orbitotomy. At surgery the mass was well circumscribed, extraconal, very firm and did not invade or adhere to other structures. The tumor was removed in toto. The diagnosis was confirmed by histopathological examination, the lesion was nodular, and there was differentiation toward the adnexal ductal epithelium with chondromyxoid and adipocytic differentiation in the stroma. No recurrence was seen with one year follow-up.

CS should be included in the differential diagnosis of intra-orbital tumors. Complete resection remains the best therapeutic option to prevent recurrence. Close followup is recommended because malignant transformation, although rare, is possible.

## Introduction

Chondroid syringoma (CS) are rare mixed tumours of sweat-gland which were first described by Billroth in 1859, that have both a bening and malignant form. Hirsch and Helwig in 1961 [1] gave them the appellation CS, because of the presence of sweat gland elements which are set in a cartilaginous stroma. The commonest sites are the scalp, cheek, nose, upper lip, chin, and the forehead.

CS of the orbit are extremely rare. In the better of our knowledege, only one case has been reported in the english litérature [2]. Here we report an unusual case of a patient who underwent complete resection of a intraorbital CS. The clinical presentation, histologycal findings and treatment, with review of the relevant literature, are discussed.

### Case report

**Figure 1:**
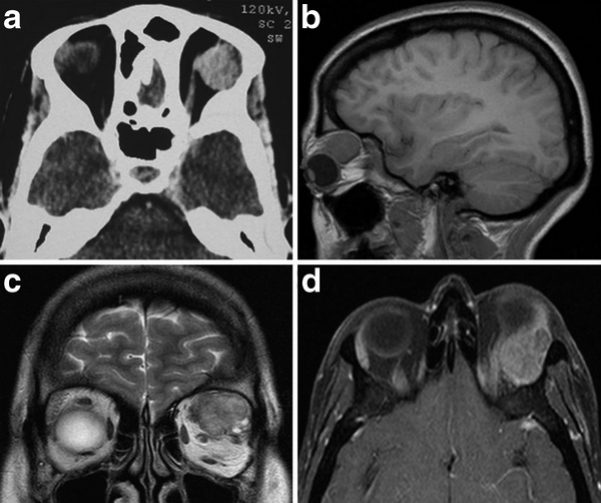
**(A): Cranial CT scan demontrated an intra-orbital tumor, well circumscribed with homogeneous enhancement without bone erosion**. (**B**, **C **and **D**): MRI showed a round mass that was extraconal, isointensity on T1-weighted imaging, slightly hyperintensity on T2-weighted imaging and enhanced after intravenous contrast administration.

**Figure 2:**
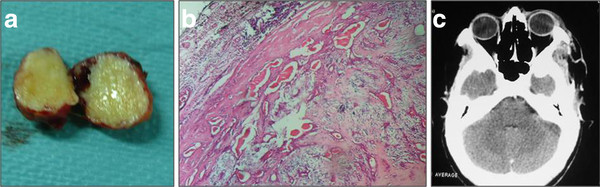
**(a): gross pathologic analysis revealed well-encapsulated tan tissue measuring 2 × 3 × 1,5 cm**. No areas of hemorrhage or necrosis were noted. (**b**): a photomicrograph of the surgical specimen shows an encapsulated proliferation of nonbranching ducts set in chondromyxoid stroma. (hematoxylin and eosin stain, ×100). (**c**): postoperative CT image schows complete removal with good orbital reconstruction.

A 41-year-old woman presented with a history of left exophtalmos for 8 years and deterioration of visual acuity in the last 8 months. His medical and ocular histories were unremarkable. Physical examination disclosed no system abnormality except ocular findings. There was an indolor left exophtalmos, no axil with downward displacement of the globe. Abduction of the left eye was slightly restricted. Partial visual field defect was noted. Visual acuity was 8/10 in the left eye and 10/10 in the right eye. Cranial CT scan demontrated a superolaterally solid tumor, well circumscribed with homogeneous enhancement without bone erosion (Figure 1A). MRI showed a round mass that was extraconal, isointensity on T1-weighted imaging, slightly hyperintensity on T2-weighted imaging and enhanced after intravenous contrast administration (Figure 1B, C and 1D). The globe was displaced anteriorly. The optic nerve was medially displaced by the intervening mass. Our preoperative diagnoses was pleomorphic adenoma and adenoid cystic carcinoma. The tumor was removed completely through lateral orbitotomy with minimal orbital rim osteotomy. At surgery the mass was extraconal, very firm, enucleated easily and schowed no invasion or adhesion to other structures, including skin, globe, extraocullar muscles, lacrimal gland, and orbital bone. The diagnosis of CS was confirmed by histopathological examination. Gross pathologic analysis revealed well-encapsulated tan tissue measuring 2 × 3 × 1,5 cm (Figure 2a). No areas of hemorrhage or necrosis were noted. Histologically, the lesion was nodular, and there was differentiation toward the adnexal ductal epithelium with chondromyxoid and adipocytic differentiation in the stroma (Figure 2b). Early postoperative CT image schows complete removal with good orbital reconstruction (Figure 2c). The postoperative course was uneventful. The patient was discharged 4 days after the operation without any complication. Eye motility, visual acuity and cosmetic appearance improved satisfactorily. No recurrence has been seen with one year of follow-up.

## Discussion

Chondroid syringoma (CS) is a rare, benign, skin appendagal tumor, first described by Billorth in 1859 for a group of tumors of the salivary gland that contained varying amounts of mucoid and cartilaginous material [3]. Virchow and Minssen referred to them as mixed tumors with both epithelial and mesenchymal origin [4]. The term « chondroid syringoma » was coined by Hirsh and Helwig (1961) to refer to a type of skin tumour originating in the apocrine sweat glands, comprised of glandular elements and stroma, similar to cartilage (mixed cutaneous tumour). They also proposed five histopathological criteria for its diagnosis: 1) nests of cuboidal or polygonal cells; 2) intercommunicating tubuloalveolar structures lined with two or more rows of cuboidal cells; 3) ductal structures composed of one or two rows of cuboidal cells; 4) occasional keratinous cysts; 5) a matrix of varying composition. Chondroid syringomas may have all five characteristics or manifest only some [1].four criterias were found in our case.

Incidence of CS has been reported as less than 0.01 percent of primary tumors of the skin [4]. CS usually affects middle-aged or older male patients [4].

Clinically, CS presents typically as a slow growing, painless, firm, non-ulcerated subcutaneous or intracutaneous nodule. The lesion commonly measures 0.5-3 cm in diameter [5]. However, larger forms of CS have been described [5].

The sites of predilection for CS are on the head and neck region, particularly cheek, nose, or skin above the lip [3,5]. Less commonly, this tumor can develop on the scalp, eyelid, hand, foot, forehead, axillary region, abdomen, penis, vulva, and scrotum [6-8].orbital location is exceptional. A medline database was questioned using the key words: CS-orbit, only one case can be found [2].

Kitazawa T and al [2] have reported in 1999 the first case of a woman complaining of left exophtalmos and diplopia, a firm mass was palpable in the left lateral canthal region, CT and MRI demonstrated a extraconal mass extending to the malar subcutis and associated with compression of the globe, the tumor was resected easily and the diagnosis of benign chondroid syringoma was confirmed by histopathological examination. No mass palpable was found in our case.

Histologically, CS consists of mixed epithelial and mesenchymal elements, with epithelial cells arranged in cords and forming tubules with a myoepithelial layer, set in a myxoid or chondroid stroma [4,9]. Osteoid stroma or mature adipocytes can be present in rare cases [9]. Two histological variants of this tumor are described, the eccrine type with smaller lumens lined by a single row of cuboidal epithelial cells and the apocrine variant with tubular and cystic branching lumina lined by two rows of epithelial cells [4]. Immunohistochemical study shows focal positivity for keratin, vimentin, desmin, and S-100 protein in the stroma [4].

Radiological features of CS are not as suggestive as the histological findings. The MRI features are non-specific, but can accurately depict the anatomic extent and identify tissue of origin, depth of invasion and relation to adjacent structures, such as muscles and bones [10].

The differential diagnosis of CS is made with other benign orbital lesions include pleomorphic adenoma, dermoids, lipomas, neurofibromas, meningioma, lymphangiomas, Cavernous hemangiomas and histiocytic tumors. [11]. We describe our patient to point out that this tumor should be considered a rare possibility in the differential diagnosis of orbital tumors. CS lesions usually are not clinically or radiology distinctive and the diagnosis is made on histological examination [3,4].

The treatment of choice is complete excision without esthetic or functional disruption of surrounding structures [4]. Lateral orbitotomy is a safe approach in the surgical management of lesions in the lateral orbital region, particularly if the lesions are extraconal [12]. It has been suggested that incisional biopsy or fine-needle aspiration may disrupt the tumor and seed local tissue, increasing the risk of recurrence. In our observation, the tumor was completely excised without recurrence within a 1-year followup.

Chondroid syringoma is a benign tumor. However, rare cases of malignant CS have been reported [3,13]. These malignant forms occur more commonly in younger female patients, and have a predilection for occurring on the trunk or extremities [3,13]. These tumors often are larger than 3 cm and are locally invasive. Histological findings such as cytologic atypia, infiltrative margins, satellite tumor nodules, tumor necrosis, and involvement of deep structures are considered as signs of malignant transformation [4,14]. Close follow-up of these tumors is recommended because of risk of malignancy and recurrence.

## Conclusion

Chondroid syringoma should be included in the differential diagnosis of intraorbital tumors, especially in middle-aged patients. Although malignant forms and recurrence are extremely rare, wide excision of the tumor and long-term followup are the most effective ways of management.

## Consent

Written informed consent was obtained from the patient for publication of this Case report and any accompanying images.

## Competing interests

The authors declare that they have no competing interests.

## Authors' contributions

HB, AA Conception and Design. HB Acquisition of Data. AA, BE, MO Analysis and Interpretation of Data. HB Drafting the Manuscript. AA Revising It for Intellectual Content. MB, BE Final Approval of the Completed Article. All authors read and approved the final manuscript.
